# Fipronil application on rice paddy fields reduces densities of common skimmer and scarlet skimmer

**DOI:** 10.1038/srep23055

**Published:** 2016-03-16

**Authors:** Atsushi Kasai, Takehiko I. Hayashi, Hitoshi Ohnishi, Kazutaka Suzuki, Daisuke Hayasaka, Koichi Goka

**Affiliations:** 1 National Institute for Environmental Studies, Onogawa 16-2, Tsukuba, Ibaraki 305-8506, Japan; 2 Faculty of Agriculture, Kindai University, Nakamachi 3327-204, Nara 631-8505, Japan

## Abstract

Several reports suggested that rice seedling nursery-box application of some systemic insecticides (neonicotinoids and fipronil) is the cause of the decline in dragonfly species noted since the 1990s in Japan. We conducted paddy mesocosm experiments to investigate the effect of the systemic insecticides clothianidin, fipronil and chlorantraniliprole on rice paddy field biological communities. Concentrations of all insecticides in the paddy water were reduced to the limit of detection within 3 months after application. However, residuals of these insecticides in the paddy soil were detected throughout the experimental period. Plankton species were affected by clothianidin and chlorantraniliprole right after the applications, but they recovered after the concentrations decreased. On the other hand, the effects of fipronil treatment, especially on Odonata, were larger than those of any other treatment. The number of adult dragonflies completing eclosion was severely decreased in the fipronil treatment. These results suggest that the accumulation of these insecticides in paddy soil reduces biodiversity by eliminating dragonfly nymphs, which occupy a high trophic level in paddy fields.

Rice paddy fields serve several important functions for biodiversity in Japan. For example, paddy fields act as alternative habitats for marsh-inhabiting aquatic organisms^1^. Japanese paddy fields occupy 24 690 km^2^ (6.5% of Japan’s land area)^2^, which is nearly twice the total area of surface water in Japan (13 400 km^2^)^3^. Paddy fields also contribute to the formation of qualitatively unique agroecosystems. Rice paddies exclude large fishes, which occupy the apex predator niche in bodies of fresh water. The conditions of Japanese paddy fields alternate between dry (in autumn and winter) and wet (in spring and summer) in synchrony with the monsoon climate of East Asia. Thus, many aquatic organisms in paddy fields need to escape the temporary dry conditions.

Dragonflies (Odonata) have been regarded as an indicator of the ecological quality of aquatic environments^4^,^5^,^6^,^7^. In environments with no large fishes, both the adults and nymphs of dragonflies occupy a relatively high trophic level^8^, and thus cause top-down effects on paddy biological communities. In addition, dragonflies have a long generation time, so their condition is likely to reflect the effects of agrochemicals on paddy agroecosystems at annual or longer time scales. Paddy fields are indispensable habitat for several dragonfly species. For example, some *Sympetrum* species [including *Sympetrum frequens* (Selys), *Sympetrum darwinianum* (Selys) and *Sympetrum infuscatum* (Selys)] use paddy fields as their main oviposition site and nymph habitat^9^,^10^.

Several reports suggested that nursery-box application of neonicotinoid insecticides and fipronil are the major causes for the decline of Japanese *Sympetrum* species since the 1990s^11^,^12^,^13^,^14^. According to standard application methods specified by insecticide manufacturers, these pesticide is applied to nursery-boxes only once before transplantation and continues to control pests for several months via systemic or slow effects. These applications to nursery-boxes in each area cause high concentration weeks, because the transplantation of rice seedlings tends to be conducted within few weeks in each area. Unfortunately, some aquatic organisms with limited migration ability, including *Sympetrum* nymphs, are not likely to be able to escape from these areas of high concentration.

Mesocosm ecotoxicity testing is a good method for investigating which species in paddy fields are affected by pesticides^15^. For example, Sánchez-Bayo & Goka^16^ and Hayasaka *et al.*^17^ reported that the aquatic communities treated with imidacloprid had lower species abundance than that of the control and another treatment (zinc pyrithione^16^; fipronil^17^) based on paddy mesocosm experiments. When assessing the effects of pesticides on communities that include non-target species, the species that will respond to pesticide application is generally unknown before the examination. To assess spontaneous responses (e.g., community succession via natural migration and oviposition) to a change of pesticide concentration, we have to design the experiment to reflect a real paddy field system. Ecotoxicity tests using a lysimeter^11^,^13^ are limited in that they can only assess the response of manually introduced species. On the other hand, in ecotoxicity tests using real paddy fields, it is technically difficult to control the various environmental factors of each paddy field properly (e.g., fauna and flora, planting history, cultivation methods). However, ecotoxicity tests using a paddy mesocosm can more accurately predict the potential pesticide impacts on the biological communities than the abovementioned tests^18^ because (1) a paddy mesocosm can naturally incorporate the migration of species that use paddy fields, (2) interspecific interactions among the species are not disturbed and (3) the physicochemical environment resembles that of a real paddy field and is still controllable.

In this study, we conducted paddy mesocosm experiments to investigate the effects of three systemic insecticides (clothianidin, fipronil and chlorantraniliprole), which are the major insecticides used for nursery-box application in Japan, on rice paddy field biological communities, and particularly their effects on Odonata. Based on our findings, we also discuss the effects of insecticide residues on paddy field biological communities.

## Results

### Exposure dynamics of the target insecticides

**Figure 1:**
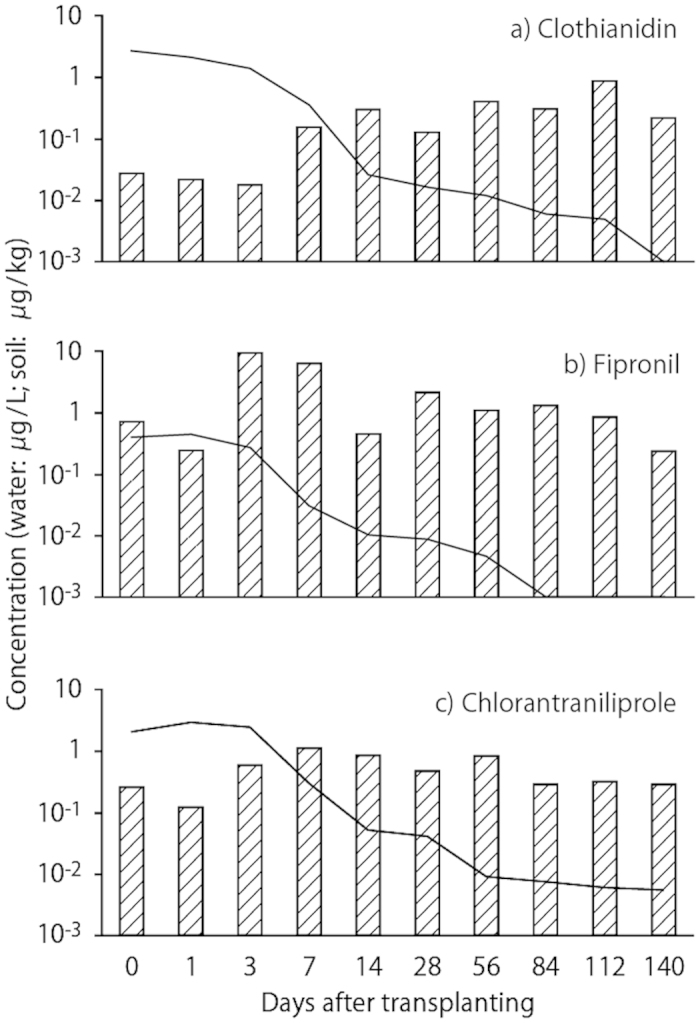
Concentration dynamics of clothianidin, fipronil and chlorantraniliprole in water (lines) and soil (bars) of paddy mesocosms.

The concentrations of clothianidin, fipronil and chlorantraniliprole in water 2 h after transplanting of the rice seedlings were 2.69, 0.394 and 2.06 μg L^−1^, respectively (Fig. 1). The maximum concentration of clothianidin in water was at 2 h after transplanting (Fig. 1a), whereas the concentrations of fipronil and chlorantraniliprole in water increased slightly after 1 day (Fig. 1b,c). The concentrations of clothianidin and fipronil in water declined to below the LOD after 20 and 12 weeks, respectively, whereas that of chlorantraniliprole remained above the LOD until the end of the monitoring period. The half-lives of these insecticides were determined using first-order kinetics. The DT_50_ values of clothianidin, fipronil and chlorantraniliprole in water were 14.4 days (*y* = −0.048*x* − 0.690, *r*^2^ = 0.75), 16.9 days (*y* = −0.041*x* − 2.36, *r*^2^ = 0.74) and 16.1 days (*y* = −0.043*x* − 0.537, *r*^2^ = 0.71), respectively.

Concentrations of clothianidin in soil increased until 112 days after transplanting and remained high until the end of the monitoring period (0.216 μg kg^−1^; Fig. 1a). Concentrations of fipronil in soil increased until 3 days after transplanting, decreased slowly until 14 days and remained fairly stable until the end of the monitoring period (0.241 μg kg^−1^; Fig. 1b). Concentrations of chlorantraniliprole in soil increased until 7 days after transplanting and then remained stable until the end of the monitoring period (0.347 μg kg^−1^; Fig. 1c). The DT_50_ of clothianidin in soil could not be calculated because it did not show a decreasing trend. The DT_50_ values of fipronil and chlorantraniliprole in soil were 40.8 days (*y* = −0.017*x* + 1.32, *r*^2^ = 0.50) and 69.3 days (*y* = −0.010*x* + 0.162, *r*^2^ = 0.71), respectively.

### Abiotic factors

In the early stage (0–21 days) the only difference in turbidity was that between the control and chlorantraniliprole treatments, and no differences in turbidity were noted among the four treatments in the middle (28–84 days) and late (98–140 days) stages (Table 1). Throughout the three stages, the only difference in pH was that between the chlorantraniliprole and clothianidin treatments in the late stage. The dissolved oxygen levels in the clothianidin treatment were lower during the early (*P* < 0.05 vs. chlorantraniliprole) and late (*P* < 0.05 vs. all others) stages.

### Effects on the abundance and diversity of organisms

A total of 55 aquatic vertebrates and invertebrates were identified to the lowest taxonomic level possible over the experimental period (Table 2). These included 53 invertebrates (10 zooplankton crustaceans, 2 oligochaeta and 41 insect species) and 2 vertebrates (1 fish and 1 amphibian species).

**Figure 2:**
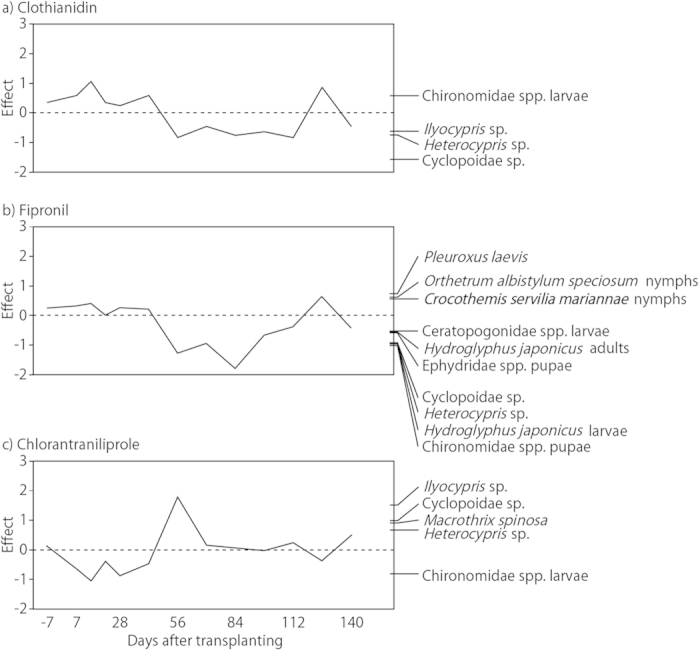
Principal response curves indicating the effect of the insecticides clothianidin, fipronil and chlorantraniliprole on aquatic assemblages of the experimental paddy mesocosms. The vertical axes (effect) show the species weight and canonical coefficient. Zero lines indicate the control treatment, and the solid lines indicate the difference in assemblage structure in response to each insecticide application. Only species with a score above 0.5 or below −0.5 are shown.

There were no significant differences between the control and clothianidin or chlorantraniliprole treatments with regard to the degree of the effect on the abundance and diversity of aquatic organisms (Monte Carlo permutation tests, *P* > 0.05, Fig. 2a,c). On the other hand, there was a significant difference between the control and fipronil treatment (Monte Carlo permutation tests, *P* < 0.05, Fig. 2b). Mainly plankton species, Cyclopoidae sp. (Cyclopoida: Cyclopoidae), *Heterocypris* sp. (Ostracoda: Podocopida) and *Ilyocypris* sp. (Ostracoda: Podocopida) were affected negatively in the early stage by clothianidin treatment (Fig. 2a); the effects on plankton species became positive after day 56 since transplanting. PRC scores for the clothianidin treatment oscillated around zero after 126 days since transplanting. In the fipronil treatment, PRC scores were small and remained near zero at first (Fig. 2b). *Pleuroxus laevis* (Sars) (Branchiopoda: Cladocera), *O. a. speciosum* (Insecta: Odonata) and *C. s. mariannae* (Insecta: Odonata) were affected negatively after day 56. Chironomidae spp. (Insecta: Diptera), *Hydroglyphus japonicus* (Gschwendtner) (Insecta: Coleoptera), *Heterocypris* sp. (Ostracoda: Podocopida), Ephydridae spp. (Insecta: Diptera) and Ceratopogonidae spp. (Insecta: Diptera) were affected positively after day 56. PRC scores of the fipronil treatment began to decline on day 126. In the early stage of the chlorantraniliprole treatment, mainly plankton species, *Ilyocypris* sp. (Ostracoda: Podocopida), Cyclopoidae sp. (Cyclopoida: Cyclopoidae), *Macrothrix spinosa* King (Branchiopoda: Diplostraca) and *Heterocypris* sp. (Ostracoda: Podocopida) were affected negatively (Fig. 2c), but the effects on plankton species changed to positive after day 56. PRC scores for the chlorantraniliprole treatment oscillated around zero after day 70. Opposite effects were noted between the abovementioned plankton species and Chironomidae spp. in the clothianidin and chlorantraniliprole treatments, whereas these taxa were affected similarly in the fipronil treatment.

**Figure 3:**
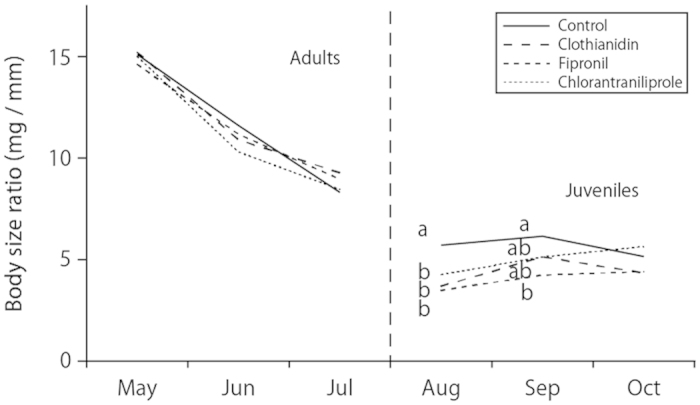
Body size ratio of adult medaka and their juveniles in the experimental paddy mesocosms throughout the entire cultivation period. Different letters indicate significant differences (*P* < 0.05, pairwise *t*-test with Holm’s correction) on the respective sampling dates.

The body size ratio of adult medaka did not differ among treatments (Fig. 3). Although the body size ratios of their juveniles right after sampling were significantly different between the control and the insecticide treatments, this difference disappeared after 2 months, except in the case of fipronil.

### Comparisons of the total number of *C. s. mariannae* and *O. a. speciosum* nymphs and exuviae

**Figure 4:**
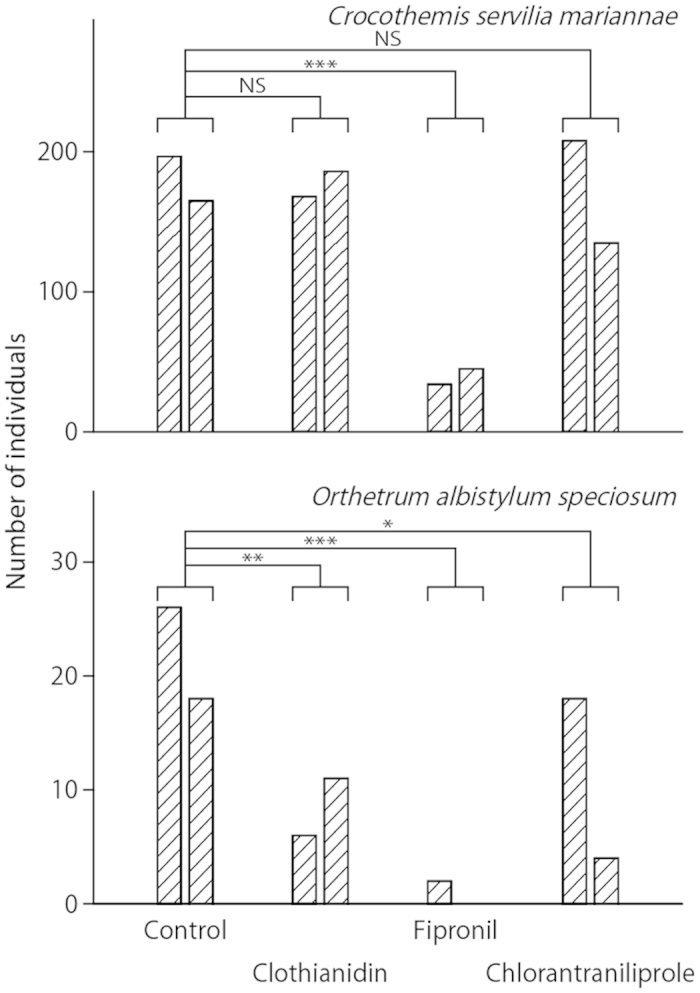
Total numbers of captured nymphs of the dragonfly species *Orthetrum albistylum speciosum* and *Crocothemis servilia mariannae* in each paddy. Each bar represents a replicate. Asterisks indicate a significant difference as compared to the control (Dunnett’s test, *P < 0.05, **P < 0.01, ***P < 0.001). “NS” indicates no significant difference.

**Figure 5:**
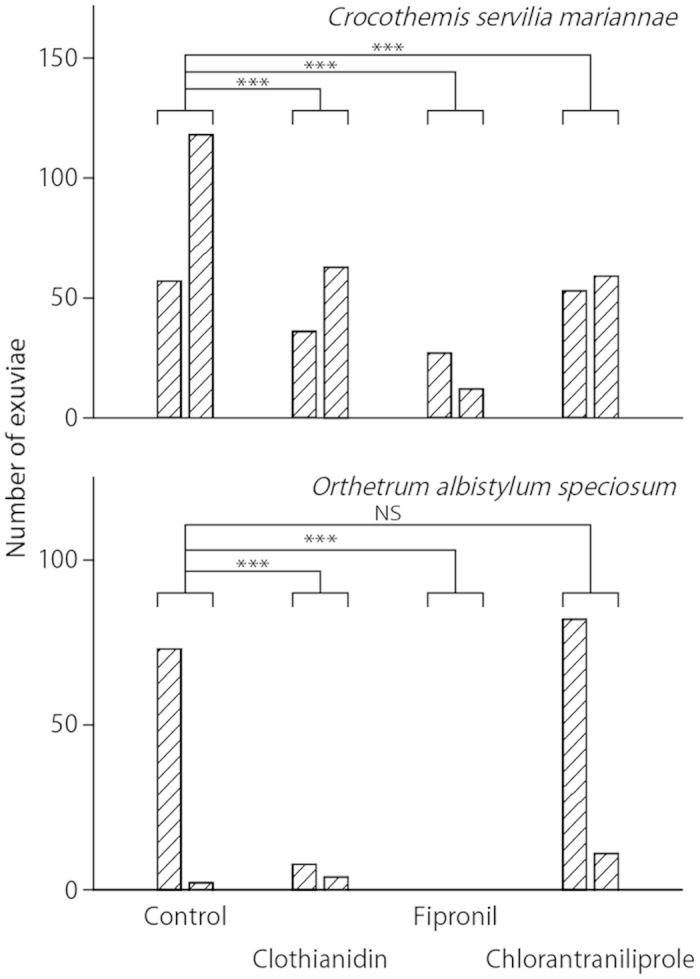
Total number of collected exuviae of the dragonfly species *Orthetrum albistylum speciosum* and *Crocothemis servilia mariannae* in each paddy. Each bar represents a replicate. Asterisks indicate a significant difference as compared with the control (Dunnett’s test, ***P < 0.001). “NS” indicates no significant difference. The number of *O. a. speciosum* exuviae in the fipronil treatment was zero in both replicates. Therefore, Dunnett’s test could not be applied because the variance in the treatment was zero. However, these values were interpreted as being significantly lower than that of the control because no exuviae in both replicates represented a larger decrease than in the significant clothianidin case.

Figure 4 illustrates the total catches of *C. s. mariannae* and *O. a. speciosum* nymphs in each paddy mesocosm. For *C. s. mariannae*, the numbers of nymphs in the clothianidin and chlorantraniliprole treatments were not significantly different from that of the control (Dunnett’s test after Poisson regression; clothianidin: *P* = 0.95; chlorantraniliprole: *P* = 0.74), whereas that in the fipronil treatment was significantly less than that of the control (*P* < 0.001). For *O. a. speciosum*, the numbers of nymphs in all the insecticide treatments were significantly less than that of the control (clothianidin: *P* = 0.0026; fipronil: *P* < 0.001, chlorantraniliprole: *P* = 0.049). Figure 5 shows the total number of *C. s. mariannae* and *O. a. speciosum* exuviae collected in each paddy mesocosm. For *C. s. mariannae*, the numbers of exuviae in all the insecticide treatments were significantly less than that in the control (clothianidin: *P* < 0.001; fipronil: *P* < 0.001, chlorantraniliprole: *P* < 0.001). For *O. a. speciosum*, the numbers of exuviae in the chlorantraniliprole treatment was not significantly different from that of control (*P* = 0.41), whereas the numbers in the clothianidin treatment were significantly different from that of the control (*P* < 0.001). The number of *O. a. speciosum* exuviae in the fipronil treatment was zero in both replicates. Therefore, Dunnett’s test could not be applied because the variance in the treatment was zero. However, these values were interpreted as being significantly lower than that of the control because no exuviae in both replicates represented a larger decrease than in the significant clothianidin case.

## Discussion

The results of this study suggest that applications of systemic insecticides have negative impacts on dragonflies, which are some of the most important species in Japanese paddy field ecosystems. The effects on these species are not being considered in the present framework of agrochemical management. Under Japan’s Agricultural Chemicals Regulation Law, for example, pesticide registration requires laboratory tests of three aquatic taxa (algae, *Daphnia magna* Straus and fish) based on the Organisation for Economic Co-operation and Development test guidelines^19^. Although these test results are relatively easy to evaluate and compare^20^,^21^,^22^,^23^, they are not suitable for assessing seasonality of the effects or the conservation of local biodiversity and endemic species because the following three factors are ignored: (1) the change in exposure that occurs in the field, which depends largely on the physicochemical characteristics of each pesticide, such as water solubility, adsorption and hydrolytic and photolytic properties^17^,^24^, (2) differences in sensitivity among the species in the field^25^ and (3) interspecific interactions within the community structure^26^. From the perspective of conserving paddy field ecosystems in Japan, it would be preferable to adopt paddy field mesocosm experiments to assess agrochemical management.

Our findings strongly suggest that fipronil application is a major cause of the sharp decline of dragonfly populations in Japanese paddy fields, in agreement with previous research^11^,^12^,^13^,^14^,^27^. Our mesocosm experiments showed that fipronil application to nursery boxes resulted in a severe decrease in the number of individuals and exuviae of two dragonfly species (Figs 4 & 5), supporting fipronil’s adverse effects on dragonflies. Because the water concentration of fipronil decreased rapidly to 0.1 μg L^−1^ within 2 weeks after application and to the LOD by 6 August (84 days after exposure), we must consider two possible scenarios: (1) fipronil has strong acute toxicity or (2) some metabolites of fipronil have strong acute toxicity. Several studies reported high toxicity of the metabolites of fipronil on aquatic organisms (e.g., crayfish^28^; midges^29^), and acute toxicity assessments of not only fipronil but also its metabolites are needed to measure their effects on dragonflies. The numbers of *O. a. speciosum* nymphs in the clothianidin and chlorantraniliprole treatments decreased significantly compared with control, whereas those of *C. s. mariannae* did not decrease. These findings reveal interspecific differences in sensitivity, highlighting the importance of performing mesocosm experiments. Thus, mesocosm enables to give the order of the sensitivity examination priority. Note that the effect of these insecticides on *Sympetrum* species could not be confirmed in this study because the occurrence of *Sympetrum* nymphs requires oviposition in the previous autumn.

The concentrations of each insecticide in the water declined rapidly in the first 2 weeks of the experiment, followed by a slow decline thereafter (Fig. 1). The initial rapid declines were mainly explained by hydrolysis or aqueous photolysis of the insecticide compounds, whereas the slower phase of decline may reflect re-emission of the compounds from the soil into the water above. These results indicate that the residues in soil affect the concentrations of insecticides in water over a long period. Hayasaka *et al.*^24^ reported that the amounts of fipronil and imidacloprid in paddy soil accumulated following multi-year applications. Although the activity of insecticides adsorbed on soil particles may be lower than that in water, multi-year applications of the insecticides used in this study might be causing unexpectedly high concentrations in real paddy fields due to insecticide accumulation. The concentration of fipronil in paddy water was lower than the LOD after day 84 since transplanting, but the effects of fipronil metabolites should be considered because some remain in rice paddy water^30^. Thus, future studies should assess the re-emission of insecticides from residuals in soil and levels of their metabolites in order to improve the conservation of local biodiversity.

Additional research is needed to understand the roles played by fipronil and other insecticides in the severe declines of dragonfly species in Japan. First, paddy mesocosm experiments incorporating the natural oviposition of *Sympetrum* species in autumn are needed to assess the impacts of fipronil on these taxa, because the severe decline of *Sympetrum* populations is a particular concern in Japan^14^. In the present mesocosms, *C. s. mariannae* and *O. a. speciosum* were present in the biological community, but no *Sympetrum* species were found because their last oviposition period occurred prior to the setup of the field plots. Second, the relationship between fipronil concentrations in the water and soil of real paddy fields and the habitats of nymphs of *Sympetrum* and other dragonfly species should be examined. Finally, studies are needed to examine the degree and variation of insecticide sensitivity of *Sympetrum* and other dragonfly species by using acute toxicity tests. The combination of these surveys will provide firm scientific evidence for managing the environmental load of fipronil and other insecticides and conserving the biodiversity in Japanese paddy fields.

## Methods

### Tested insecticides

The systemic insecticides clothianidin, fipronil and chlorantraniliprole were tested. Their physicochemical and acute toxicity data are given in Table 3. Clothianidin, which belongs to the chloronicotinyl chemical family, is one of the most widely used insecticides on the global market. Fipronil, which belongs to the phenylpyrazole chemical family, is also used frequently because it has a broad insecticidal spectrum. Although there are few reports on the amount of fipronil applied, the shipping amount of fipronil in Japan (32 221 kg in 2012) is on the same order of magnitude of that of imidacloprid (64 886 kg in 2012), clothianidin (66 687 kg in 2012) and thiamethoxam (36 796 kg in 2012)^31^. The use of clothianidin and fipronil was banned by the European Union in December 2013 due to the potential risk to bees^32^. The application of chlorantraniliprole, which belongs to the anthranilic diamide chemical family, on Japanese paddy field has expanded rapidly in recent years, with only 166 kg shipped in 2009, versus 33 216 kg in 2012^31^.

### Experimental design

In this study, we used experimental design and monitoring methods similar to those used in the paddy mesocosm study of Sánchez-Bayo & Goka^16^. Eight independent paddies (4.0 m length × 1.7 m width), consisting of four treatments (clothianidin-, fipronil- or chlorantraniliprole-treated field and no chemical treatment as the control) with two replicates, were set up at the National Institute for Environmental Studies, Tsukuba Prefecture, Japan (36°02′N, 140°07′E). The fields are not connected to each other. Sediment and seed banks from uncontaminated areas surrounding the study site were spread on the experimental paddies 1 month before the transplanting of rice seedlings. The experimental paddies were flooded with bore water to a depth of approximately 5 cm on 22 April 2013, and then maintained in that condition for 23 days in order to promote the natural development of aquatic assemblages. Paddies were then irrigated with agricultural groundwater (24 mm day^−1^). The depth of paddy water was maintained by using an overflow system until the end of the experiment. The paddies were planted with rice seedlings (cv. Nipponbare) on 14 May 2013 using an array of 21 × 6 bunches per field, following standard cultivation practices in the region.

At 24 h before transplanting, nursery boxes of rice seedlings (58 cm internal length × 28 cm internal width × 2.8 cm internal depth) were treated with Dantotsu^®^ (0.50% granular clothianidin, lot no. 15.10-OAB597, Sumitomo Chemical Co., Ltd., Tokyo, Japan), Prince^®^ (1.0% granular fipronil, lot no. 16.10-NJ119, Nissan Chemical Industries, Inc., Tokyo, Japan) or Ferterra^®^ (0.75% granular chlorantraniliprole, lot no. 15.10-OA323, DuPont Kabushiki Kaisha, Tokyo, Japan), at a rate of 50 g per box, which is an average recommended rate of application on commercial rice fields.

The abiotic factors of turbidity, pH and dissolved oxygen in the fields were measured on every survey date. Turbidity was measured using a turbidity meter (HI-93703C; HANNA Instruments Japan, Tokyo, Japan), and pH and dissolved oxygen were measured by a portable multi-meter (DM-32P; DKK-TOA Corporation, Tokyo, Japan).

### Chemical analysis

Samples of water (250 mL) and surface soil (2–3 cm depth, 250 g) were taken 2 h after transplanting the rice seedlings (day 0) and then on days 1, 3, 7, 14, 28, 56, 84, 112, and 140 from three random sampling points in each insecticide-treated field. To avoid photolysis and degradation after sampling, the samples were collected in amber bottles sealed by Parafilm M^®^ (Nikkei Products Co., Ltd., Osaka, Japan) and stored in a freezer (−28 °C) until analysis.

Residue concentrations of the three insecticides in water and soil were analysed at the certified analytical laboratories of Heiseiriken Co., Ltd. (Utsunomiya, Tochigi, Japan). The method of Baskaran *et al.*^33^ with slight modification (omission of liquid-liquid extraction on water samples; omission of concentration using rotary evaporator on soil samples) was followed for extraction of water and soil residues of clothianidin, fipronil and chlorantraniliprole. Water samples were filtered using centrifugal filtration units (Millipore Ultrafree 0.20 μm, Merck Millipore Corporation, Darmstadt, Germany). Filtrates were analysed by liquid chromatography tandem mass spectrometry (Xevo-TQ, Waters Corporation, Milford, MA, USA). The soil samples were air-dried in a dark room. All extractions were performed at room temperature. About 10 g of soil samples from the clothianidin and chlorantraniliprole treatments were extracted with 10 mL of distilled water, 10 mL of acetonitrile and 4 g of NaCl while being stirred on a reciprocating shaker (180 rpm, 60 min). About 10 g of soil samples from the fipronil treatment were extracted with 10 mL of acetone-water (90:10, v/v) while being stirred on a reciprocating shaker (180 rpm, 60 min). After extraction, solutions were centrifuged for 10 min at 3000 rpm. The extract after percolation (Millipore Ultrafree 0.20 μm) was analysed by liquid chromatography tandem mass spectrometry (Xevo-TQ). The limits of detection (LOD) in water and soil samples of each insecticide were 0.001 μg L^−1^ and 0.06 μg kg^−1^, respectively.

### Census and sampling of aquatic assemblages

The insecticide-treated mesocosms and controls were monitored every 2 weeks from 7 May to 2 October 2013. For the sake of estimating the insecticides’ effects on fish, 10 adult male and 10 adult female medaka [*Oryzias latipes* (Temminck et Schlegel)] were released in each mesocosm on the transplanting day (13 May 2013) and allowed to reproduce. Among the medaka released, 5 males and 5 females were enclosed in a plastic 2.1-mm-mesh net (300 mm × 300 mm × 150 mm) to allow long-term monitoring of the same individuals unimpeded by the effects on the growth of aquatic plants and algae. The growth of medaka was measured using a body size ratio (body weight/length). After day 84, 10 juvenile fish were sampled randomly from each mesocosm and moved into the plastic nets, and then measured for the same parameters until the end of the experiment.

Organisms were sorted into three communities: (1) zooplankton; (2) benthos, including bottom-dwelling larvae, worms and molluscs and (3) aquatic arthropods, including surface-dwelling and water-borne organisms and their larvae, and medaka fish. Sampling of zooplankton, benthic organisms and aquatic arthropods was carried out in the same way as described by Hayasaka *et al.*^17^,^24^. To sample zooplankton, water (1 L) was taken from four random sampling points in each mesocosm, and after filtration through a 250-μm plankton net specimens were counted using a stereoscopic microscope (MZ16, Leica Microsystems, Wetzlar, Germany). Benthic organisms and crawling larvae were sampled using a polyvinyl chloride core (5 cm height, 10 cm diameter) that was firmly inserted to a depth of 5 cm in the sediment at three random sampling points in each mesocosm; specimens were washed through an 850-μm sieve and counted by visual observation. Aquatic organisms were sampled by scooping into a 1.6-mm-mesh between the rice seedlings in the same routes (about 10 meters), screened the aquatic animals by visual observation and counted using a stereoscopic microscope (SZ61, Olympus Corporation, Tokyo, Japan). To assess the occurrence of adult dragonflies, all the dragonfly exuviae in each mesocosm were collected for identification once a week from 14 August to 1 October 2013.

### Statistical analyses

Differences in abiotic factors (turbidity, pH and dissolved oxygen) among the four treatments were analysed by pairwise *t*-test with Holm’s correction. Abiotic data were divided into three lots corresponding to the three stages of the cultivation period (i.e., early, middle and late). Differences in medaka body size ratios among the treatments at each sampling date were also analysed by pairwise *t*-test with Holm’s correction. The response of all assemblages to the applied insecticides was analysed by principal response curves (PRCs)^34^. Statistical significance of the PRC model, in terms of displayed treatment variance, was tested by Monte Carlo permutation tests. For this analysis, the species abundances were ln (10*x* + 1) transformed in order to down-weight high abundance values^35^. Then, the significance of the PRC results was tested by Monte Carlo permutation tests; a significant difference in PRC scores between the control and insecticide-treated mesocosms indicated a large deviation from the normal aquatic assemblage composition and structure due to insecticide application. Comparisons of the total numbers of captured *Crocothemis servilia mariannae* Kiauta and *Orthetrum albistylum speciosum* (Uhler) nymphs and exuviae were analysed using Dunnett’s test after Poisson regression for multiple comparisons of the mean with a control. The software package R, version 3.1.1^36^ was used for all statistical analyses. PRC analyses were made using the “vegan” library, version 2.3-2^37^. Multiple comparisons were made using the “multcomp” library, version 1.4-1^38^.

## Additional Information

**How to cite this article**: Kasai, A. *et al.* Fipronil application on rice paddy fields reduces densities of common skimmer and scarlet skimmer. *Sci. Rep.* **6**, 23055; doi: 10.1038/srep23055 (2016).

## Figures and Tables

**Table 1:** Changes in water quality parameters (±SE) of the experimental paddy mesocosms.

Treatment	Control	Clothianidin	Fipronil	Chlorantraniliprole
Early stage (0–21 days)				
Turbidity (FTU)†	709 ± 171 a*	408 ± 78.9 ab	224 ± 70.8 ab	294 ± 51.6 b
pH	8.22 ± 0.119	8.04 ± 0.0730	8.20 ± 0.0688	8.31 ± 0.0968
Dissolved oxygen (mg L^−1^)	6.74 ± 0.448 ab	6.16 ± 0.225 a	6.84 ± 0.239 ab	7.60 ± 0.309 b
Middle stage (28–84 days)				
Turbidity (FTU)	29.3 ± 7.33	38.6 ± 8.62	24.3 ± 5.06	33.7 ± 7.22
pH	9.06 ± 0.0960	9.74 ± 0.126	9.59 ± 0.101	9.40 ± 0.117
Dissolved oxygen (mg L^−1^)	11.8 ± 0.588	11.9 ± 0.858	11.2 ± 0.594	12.3 ± 0.782
Late stage (98–140 days)				
Turbidity (FTU)	3.49 ± 1.17	2.58 ± 0.797	1.55 ± 0.288	2.29 ± 0.861
pH	8.88 ± 0.127 ab	8.62 ± 0.133 a	8.86 ± 0.0986 ab	9.08 ± 0.0854 b
Dissolved oxygen (mg L^−1^)	11.3 ± 0.726 a	8.72 ± 0.768 b	11.9 ± 0.677 a	12.3 ± 0.693 a

^*^Different letters indicate significant differences among the four treatments according to pairwise *t*-test with Holm’s correction.

^†^FTU: formazin turbidity unit.

**Table 2:** Differences in taxa composition among the experimental paddies.

	Control	Clothianidin	Fipronil	Chlorantraniliprole
Crustacea				
Cladocera	3	3	3	3
Copepoda	1	2	1	2
Ostracoda	4	4	4	4
Branchiopoda	1	1	1	1
Oligochaeta				
Tubificidae	1	1	2	2
Insecta				
Ephemeroptera	1	1	1	1
Odonata	5	4	5	5
Hemiptera	4	4	5	4
Coleoptera	8	5	7	6
Diptera	4	5	4	5
Lepidoptera	1	0	0	0
Amphibia				
Anura	1	1	1	1
Actinopterygii				
Beloniformes*	1	1	1	1
Total	35	32	35	35

^*^Adult and juveniles of medaka (*Oryzias latipes*) released in each mesocosm on the transplanting day.

**Table 3:** Physicochemical properties and acute toxicity of clothianidin, fipronil and chlorantraniliprole.

	Clothianidin	Fipronil	Chlorantraniliprole
Physicochemical properties			
Water solubility at 20 °C (mg L^−1^)	327*	3.78*	1,023*
Octanol: water partition coefficient (logPow)	0.7 (25 °C)*	4.0 (20 °C)*	2.76*
Hydrolysis half-life at 25 °C (days)	548*	>100‡	Stable††
Aqueous photolysis half-life at 25 °C (days)	0.028–0.040*	0.33‡	0.37*
Half-life in soil (days)	770†	83–200¶	4.5‡‡
Sorption in soil (Koc)	90.0–250*	542–1176§	100.1–526*
Acute toxicity			
Crustaceans (48 h LC_50_: μg L^−1^)			
*Daphnia magna*	40,000*	190*	11.6*
Fish (96 h LC_50_: μg L^−1^)			
*Lepomis macrochirus* (bluegill)	>120,000*	25–83**	>15,100††
*Oncorhynchus mykiss* (rainbow trout)	>100,000*	39–246**	>13,800††

^*^Data from Japan Plant Protection Association^39^.

^†^Data from Health Canada Pest Management Regulatory Agency^40^.

^‡^Data from Kollman & Segawa^41^.

^¶^Data from Masutti & Mermut^42^.

^§^Data from Ying & Kookana^43^.

^**^Data from United States Environmental Protection Agency^44^ ECOTOX database (http://cfpub.epa.gov/ecotox/)

^††^Data from United States Environmental Protection Agency^45^.

^‡‡^Data from Newhart^46^.
